# Emotional Creativity Improves Posttraumatic Growth and Mental Health During the COVID-19 Pandemic

**DOI:** 10.3389/fpsyg.2021.600798

**Published:** 2021-03-03

**Authors:** Hong-Kun Zhai, Qiang Li, Yue-Xin Hu, Yu-Xin Cui, Xiao-Wei Wei, Xiang Zhou

**Affiliations:** Department of Social Psychology, Nankai University, Tianjin, China

**Keywords:** emotional creativity, post-traumatic growth, mental health, perceived social support, regulatory emotional self-efficacy, COVID-19 crisis

## Abstract

Emotional creativity refers to a set of cognitive abilities and personality traits related to the originality of emotional experience and expression. Previous studies have found that emotional creativity can positively predict posttraumatic growth and mental health. The outbreak of coronavirus disease 2019 (COVID-19) has posed great challenges to people’s daily lives and their mental health status. Therefore, this study aims to address the following two questions: whether emotional creativity can improve posttraumatic growth and mental health during the COVID-19 pandemic and how it works. To do this, a multiple mediation model has been proposed, which supposes that emotional creativity is associated with posttraumatic growth and mental health through perceived social support and regulatory emotional self-efficacy. The study involved 423 participants from multiple regions with different COVID-19 involvement levels. Participants were asked to complete a questionnaire with six parts, which included Emotional Creativity Inventory (ECI), Regulatory Emotional Self-Efficacy Scale (RES), Stress-Related Growth Scale-Short Form (SRGS-SF), Multidimensional Scale of Perceived Social Support scale (MSPSS), Brief Symptom Inventory-18 scale (BSI-18), and COVID-19-related life events questionnaire. Path analysis used to examine the mediation model indicated that under the control of COVID-19-related life events and age, perceived social support mediated a positive association between emotional creativity and posttraumatic growth as well as a negative association between emotional creativity and all mental health problems, including somatization, depression, and anxiety. Regulatory emotional self-efficacy mediates the association between emotional creativity and posttraumatic growth, emotional creativity and anxiety, and emotional creativity and depression. The results suggest that emotional creativity plays an important role in coping with stressful events related to COVID-19. Furthermore, these results might provide a better understanding of the possible paths through which emotional creativity is related to psychological outcomes, such as mental health and posttraumatic growth.

## Introduction

The outbreak of the coronavirus disease 2019 (COVID-19) pandemic in early 2020 severely impacted people around the world. Statistics showed that, as of August 18, 2020, COVID-19 infected more than 22 million people worldwide and caused nearly 778,000 deaths (Wordometer, 2020). According to the estimates of the Asian Development Bank, the global economic losses caused by the COVID-19 pandemic could be as high as 8.8 trillion United States dollars (Xinhua Net, 2020). Outbreaks of infectious diseases are often accompanied by panic and worry across society. It was shown that, during the outbreaks of severe acute respiratory syndrome (SARS) in 2003 and Ebola in 2018, both medical staff and general public were susceptible to increased risks of mental health problems (Wang et al., 2003; Waterman et al., 2018). Some researchers also found that public health emergency events did harm individual mental health (Yi et al., 2010; Li et al., 2017). In addition, apart from the impact of the pandemic itself on individuals, a series of measures taken during the pandemic (such as isolation, quarantine, restriction, etc.) also have a certain impact on individual psychology. Studies have shown that restriction has negative psychological effects on both individual and interpersonal levels, and the anxiety and depression levels of restricted residents are significantly higher than those of unrestricted residents (Dong et al., 2020; Zhang D. et al., 2020).

At present, there is sufficient evidence showing that the COVID-19 pandemic has a considerable negative impact on individual mental health (Tang and Ying, 2020; Wang et al., 2020; Wu and Wei, 2020; Zhang et al., 2020; Zheng et al., 2020; Zhou, 2020). Previous studies have indicated that emotional creativity can have a negative impact on mental health problems and a positive impact on posttraumatic growth (Orkibi and Ram-Vlasov, 2019). Therefore, this study aims to answer two questions: (1) whether emotional creativity weakens the adverse effects of the pandemic on individual mental health and improves posttraumatic growth during the COVID-19 crisis; and (2) if the previous assumption is reasonable, we will try to determine the mechanisms of emotional creativity’s impact on psychological problems.

### Posttraumatic Growth

Major disasters or crises are often associated with negative psychological effects; however, negative life events also imply the possibility of growth. As Mencius (2006) (ca. 250 B.C.E./2006) said, “When God is about to place a great responsibility on a great man, the first thing he will do is to frustrate his spirit and will, exhaust his muscles and bones, expose him to starvation and poverty, and harasses him by troubles and setbacks so as to stimulate his spirit, toughen his nature and enhance his abilities.” Posttraumatic growth (PTG) refers to the positive psychological changes that individuals experience through a struggle with traumatic events or situations (Tedeschi and Calhoun, 2004). In addition, stress-related growth, benefit-finding, perceived benefit, changes in outlook, and psychological thriving can also represent the abovementioned positive psychological changes (Linley and Joseph, 2004). According to some researchers, a variety of stress events, including diseases, such as SARS and HIV/AIDS, can trigger PTG (Tu and Guo, 2010).

Emotional factors are one of the most important factors influencing PTG. Many studies have found a positive correlation between positive emotions and PTG and a negative correlation between negative emotions and PTG (Norlander et al., 2005; Thornton and Perez, 2006; Wang et al., 2011). In addition, the related process of emotional processing can also promote PTG. Manne et al. (2004) posited that emotional expression and emotional processing can significantly predict the PTG of breast cancer patients and their partners; Yu et al. (2014) have professed that positive affection, expressive revealing, and general self-efficacy are important predictors of perceived PTG of cancer survivors; Mo et al. (2013) have found that positive emotions, cathartic regulations, and self-efficacy of tumor patients can better predict their PTG; and Zhou et al. (2019) found that emotional expression and cognitive reassessment jointly mediate the predictive effect of empathy on PTG.

### Emotional Creativity

The concept of emotional creativity originates from the social construction theory of emotion (Averill and Thomas-Knowls, 1991). This theory holds that emotions can be defined as socially constituted syndromes or transitory social roles (Averill, 1980). In daily life, people’s emotional activities represent the behavior response patterns they follow in specific situations and are prescribed by society (Qiao, 2003). On this basis, Averill (1999, 2000, 2009) proposed the concept of emotional creativity, arguing that emotional creativity is the ability to experience and express emotions, and it should include three components: preparedness, novelty, and a combination of effectiveness and authenticity. Preparedness indicates that an individual who attaches importance to emotions has the willingness to think about, understand, and explore emotions and is sensitive to them. Novelty means that the emotional response is novel and unique compared to the individual’s past or social expectations. Effectiveness means that the change is helpful in solving emotional problems, and in the long run, it would be beneficial not only to the individual but also the society. Authenticity means that the emotional response should be self-expression rather than a mirror image of others’ expectations.

Averill (1999) pointed out that participants with high levels of emotional creativity, who are more confident in their abilities, tend to choose coping strategies that emphasize self-control, planned problem-solving, seeking social support, and positive re-evaluation and benefit more from loneliness than participants with low emotional creativity. Moreover, existing studies have shown that emotional creativity is positively correlated with mental health (Lattifian and Delavarpour, 2012). Oriol et al. (2016) stated that university students with high emotional creativity were more likely to experience positive emotions, such as love, gratitude, and hope. Previous studies have shown that emotional creativity is essential for individual emotion regulation (Trnka et al., 2020). Frolova and Novoselova (2015) proposed that emotional creativity can provide response flexibility in stressful situations and help transform a familiar and stereotyped emotion into other emotions. Moreover, they believed that emotional creativity could help individuals cope with unfavorable circumstances (Frolova and Novoselova, 2015).

### Perceived Social Support

Perceived social support is a kind of subjective social support that refers to an individual’s subjective feelings of being supported and understood (Sarason et al., 1991). According to the buffer theory, perceived social support, as an individual protection mechanism, can buffer the negative impact of negative stimulation on individuals, avoid negative emotions, and protect individuals’ physical and mental health (Aneshensel and Stone, 1982; Etzion, 1984; Fried and Tiegs, 1993). The buffer theory also suggests that social support may play two roles in buffering the negative impact of negative stimulation on individuals. First, social support can weaken the appraisal of negative stimulation to the individual, causing the individual to not regard some potential stimuli as stressors. Second, for negative stimuli that have been evaluated as stressors, social support can reduce stress responses of individuals when facing stress (Cohen and Wills, 1985). That is, when facing negative stimulation, individuals who perceive higher social support think they may obtain sufficient coping resources from others to reduce the appraisal of the stressor or the stress response to the stressor (Cohen and Wills, 1985). Some studies have shown that when individuals are in stressful situations, those with high perceived social support have stronger self-efficacy and better response to stress, while those with low perceived social support have weaker self-efficacy and poorer response to stress, suffering more negative emotional experiences and psychological problems (Etzion, 1984; Guo et al., 2017). Other studies have also found that perceived social support can negatively predict individuals’ depression levels (Thorsteinsson et al., 2013). Moreover, it has been shown that the PTG of cancer patients can also be significantly predicted by perceived social support (Romeo et al., 2019; Long and Wen, 2020).

### Regulatory Emotional Self-Efficacy

Regulatory emotional self-efficacy refers to the degree of an individual’s self-confidence in whether he or she can effectively regulate his or her emotional state (Bandura et al., 2003). Some studies have pointed out that there is a high correlation between self-efficacy in regulating negative emotions and depression tendency (Bandura et al., 2003), whereas self-efficacy in managing desperation and pain is negatively correlated with anxiety, depression, shyness, and loneliness and positively correlated with self-esteem and positivity (Caprara et al., 2008; Hai et al., 2019). Other studies have shown a positive correlation between regulatory emotional self-efficacy and emotional creativity (Wang and Yan, 2017).

### This Study

As with the studies discussed above, there was a positive association between emotional creativity and PTG and a negative association with mental health problems (Orkibi and Ram-Vlasov, 2019). Furthermore, Averill (1999) proposed that individuals with high emotional creativity tend to have more confidence in their ability and are more likely to seek social support in the face of stress events. According to the buffer theory, social support can protect individuals from stressful events (Aneshensel and Stone, 1982; Etzion, 1984; Cohen and Wills, 1985; Fried and Tiegs, 1993). Therefore, we hypothesized that perceived social support and regulatory emotional self-efficacy might be mediating variables in the relationship between emotional creativity and PTG as well as the relationship between emotional creativity and mental health. Besides, we also noticed that the preliminary studies showed a positive correlation between regulatory emotional self-efficacy and emotional creativity (Wang and Yan, 2017) and a correlation between regulatory emotional self-efficacy and mental health (Bandura et al., 2003; Caprara et al., 2008; Hai et al., 2019). In conclusion, the existing studies support the possible relationship between emotional creativity and posttraumatic outcomes (PTG and mental health problems) under stress events and the mediating role of regulatory emotional self-efficacy and perceived social support.

However, previous studies were mostly based on early traumatic events or specific patients (mainly cancer patients). Some studies have even suggested that cancer, as a trigger for PTG, is unique compared with other cases (Mehnert and Koch, 2007). Therefore, it is still necessary to test whether the theoretical framework applies to the trauma caused by the COVID-19 pandemic. Furthermore, we put forward the following two assumptions: (1) emotional creativity can predict PTG positively and mental health problems negatively, and (2) regulatory emotional self-efficacy and perceived social support play intermediary roles in this process.

## Materials and Methods

### Participants and Procedure

This research adopted the convenience sampling method. We recruited online voluntary participants over 18 years of age. Participants were 157 men and 282 women (total 439 participants) ranging from 18 to 51 years (*M* = 24.96; SD = 6.07) from multiple regions with varying risk levels of COVID-19 in China. They were predominantly college students (54.21%) and employed people (44.87%), with the other groups accounting for only 0.91%. Participants were asked to complete an online questionnaire that assessed their emotional creativity, regulatory emotional self-efficacy, PTG, social support, mental health, and COVID-19-related life events. All participants received a small payment (¥10) for their participation. In addition, this study was approved by the Institutional Review Board of Psychology of Nankai University, and informed consent was obtained from the participants before the experiment.

### Measures

#### Emotional Creativity

The Emotional Creativity Inventory (ECI; Averill, 1999) was used to assess emotional creativity in this study. ECI is a five-point Likert scale ranging from 1 (not at all true) to 5 (very true) that includes 26 items (e.g., “I can experience a variety of different emotions at the same time” and “My emotions are almost always an authentic expression of my true thoughts and feelings”). In this study, we used the Chinese version of ECI, which has been validated in a Chinese population (Wang and Yan, 2017). Further, the ECI scale demonstrated acceptable reliability (Cronbach’s alpha = 0.856, composite reliability = 0.871); the confirmatory factor analysis (CFA) based on structural equation modeling (SEM) indicated that ECI has acceptable constructive validity [χ^2^/*df* = 2.781, comparative fit index (CFI) = 0.793, incremental fit index (IFI) = 0.795, goodness-of-fit (GFI) = 0.872, root mean square error of approximation (RMSEA) = 0.063].

#### Regulatory Emotional Self-Efficacy

The Regulatory Emotional Self-Efficacy Scale (RES; Caprara et al., 2008) was used to assess regulatory emotional self-efficacy. RES is a five-point Likert scale ranging from 1 (not at all true) to 5 (very true) that consists of 12 items (e.g., “When others keep giving me a hard time, I can avoid getting upset”). The Chinese version used in the study has been validated in a Chinese population (Zhang et al., 2010). In addition, the RES scale has demonstrated acceptable reliability (Cronbach’s alpha = 0.774, composite reliability = 0.797), and the CFA based on SEM indicated that RES has acceptable constructive validity (χ^2^/*df* = 2.980, CFI = 0.898, IFI = 0.899, GFI = 0.945, and RMSEA = 0.067).

#### Posttraumatic Growth

The Stress-Related Growth Scale-Short Form (SRGS-SF; Cohen et al., 1998) was used to measure PTG. SRGS-SF, ranging from 0 (not at all) to 2 (a great deal), consists of 15 items (e.g., “I learned to be nicer to others” and “I learned that I want to have some impact on the world”). The Chinese version of the SRGS-SF we used has been validated in a Chinese population (Li et al., 2018). Furthermore, SRGS-SF has demonstrated acceptable reliability in this study (Cronbach’s alpha = 0.789, composite reliability = 0.798); the CFA based on SEM indicated that the SRGS-SF has acceptable constructive validity (χ^2^/*df* = 3.130, CFI = 0.817, IFI = 0.820, GFI = 0.926, and RMSEA = 0.069).

#### Perceived Social Support

The Multidimensional Scale of Perceived Social Support (MSPSS; Zimet et al., 1988; Dahlem et al., 1991) was used to assess perceived social support. MSPSS, ranging from 1 (strongly disagree) to 7 (strongly agree), includes 12 items (e.g., “I can talk about my problems with my friends” and “My family is willing to help me make decisions”). The Chinese version of the MSPSS has been validated in the Chinese population (Huang et al., 1996). Moreover, the MSPSS scale demonstrated acceptable reliability (Cronbach’s alpha = 0.900, composite reliability = 0.905), and the CFA based on SEM indicated that the MSPSS has acceptable constructive validity (χ^2^/*df* = 3.423, CFI = 0.955, IFI = 0.956, GFI = 0.948, and RMSEA = 0.074).

#### Mental Health

The Brief Symptom Inventory-18 scale (BSI-18; Derogatis, 2000, cited in Derogatis and Fitzpatrick, 2004) was used to assess mental health in this study. BSI-18, a five-point Likert scale ranging from 0 (not at all) to 4 (extremely), includes items on three dimensions: anxiety (ANX; e.g., “Feeling tense or keyed up”), depression (DEP; e.g., “Feeling no interest in things”), and somatization (SOM; e.g., “Faintness or dizziness”). In this study, we used the widely adopted Chinese version of the BSI-18 in the Chinese population (Yang et al., 2012; Huang et al., 2019). In addition, the BSI-18 scale and its three subscales (ANX, DEP, and SOM) have demonstrated acceptable reliability in this study (Cronbach’s alphas = 0.972, 0.936, 0.924, and 0.937; composite reliability = 0.975, 0.936, 0.925, and 0.939, respectively), and the CFA based on SEM indicated that BSI-18 has acceptable constructive validity (χ^2^/*df* = 3.642, CFI = 0.955, IFI = 0.955, GFI = 0.893, and RMSEA = 0.077).

#### COVID-19-Related Life Events

The COVID-19-related life events questionnaire was used to measure stressors associated with the COVID-19 pandemic. This questionnaire was adapted from SARS-related life events questionnaire (Xu et al., 2005). The COVID-19-related life events questionnaire, a five-point Likert scale ranging from 0 (Not at all) to 4 (Extremely), consists of 17 items (e.g., “Parents or other close family members died due to COVID-19” and “Loss of long-term income sources due to the pandemic, such as corporate closure or layoffs”). Moreover, the COVID-19-related life events questionnaire demonstrated acceptable reliability in this study (Cronbach’s alpha = 0.931; composite reliability = 0.946); the CFA based on SEM indicated that the COVID-19-related life events questionnaire has acceptable constructive validity (χ^2^/*df* = 3.569, CFI = 0.947, IFI = 0.947, GFI = 0.894, and RMSEA = 0.076).

### Data Analysis

We used R 3.6.0 and the lavaan package for R to analyze the collected data (Rosseel, 2012). Pearson correlations were used to analyze the relationships between stressors, EC, PSS, RES, and posttraumatic outcomes, and path analysis was adopted to establish the mediation model between variables. To test the mediating effect of perceived social support and regulatory emotional self-efficacy, the non-parametric percentile bootstrap method (Fang et al., 2011) was used to test the statistical significance of the effects in this study.

## Results

### Descriptive Statistics and Correlations

Descriptive statistics and Pearson correlations of the variables are presented in Table 1. As expected, the emotional creativity of participants was positively and significantly correlated with their perceived social support, regulatory emotional self-efficacy, and PTG [*r*(439) = 0.532, 0.670, and 0.474, respectively; *p*s < 0.001]. In contrast, the emotional creativity of participants was negatively and significantly correlated with their mental health problems, including anxiety, depression, somatization, and other symptoms [*r*(439) = −0.146, −0.199, −0.157, and −0.175, respectively; *p*s < 0.01]. These results suggest that emotional creativity might be a protective factor for mental health problems while making people grow from the COVID-19 pandemic.

**TABLE 1:** Descriptive statistics and correlation matrix of the variables (*N* = 439).

	*M*	SD	STR	EC	PSS	RES	PTG	ANX	DEP
STR	23.075	14.677							
EC	96.469	11.363	0.056						
PSS	64.916	10.019	0.002	0.532***					
RES	45.893	5.650	0.027	0.670***	0.521***				
PTG	21.658	4.615	−0.091	0.474***	0.469***	0.458***			
ANX	7.216	6.525	0.508***	−0.146**	−0.357***	−0.220***	−0.300***		
DEP	6.431	6.247	0.513***	−0.199***	−0.369***	−0.254***	−0.330***	0.870***	
SOM	5.401	6.130	0.529***	−0.157***	−0.307***	−0.190***	−0.270***	0.844***	0.863***

STR, COVID-19-related life events (stressor); EC, emotional creativity; PSS, perceived social support; RES, regulatory emotional self-efficacy; PTG, posttraumatic growth; ANX, anxiety; DEP, depression; SOM, somatization, the same below. **p < 0.01, ***p < 0.001.

### Mediation Analysis

**FIGURE 1:**
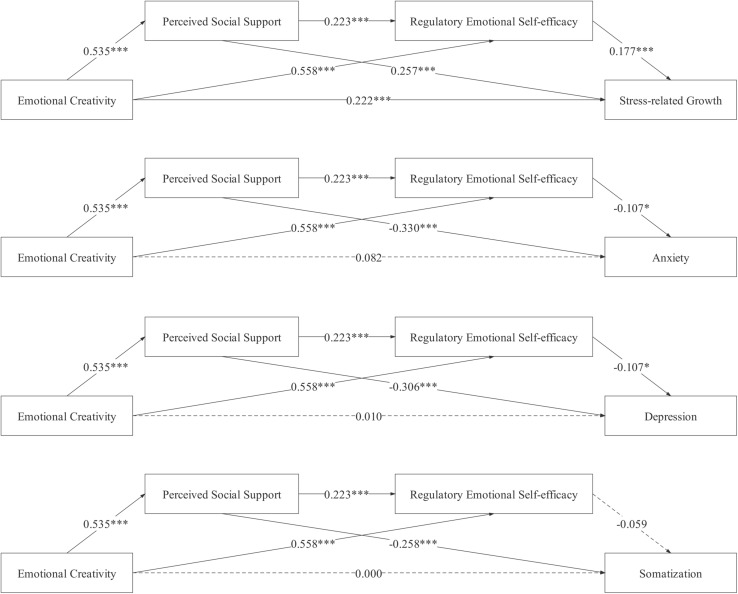
Results for the mediation model (all estimates are under the control of COVID-19 related life events and age).

Path analysis was used to test the hypothesized mediation model. Scores from all variables were converted to *z*-scores before the analysis. The coefficient of each path was significant under the control of the COVID-19-related life events and age, except emotional creativity on anxiety, depression, somatization, and regulatory emotional self-efficacy on somatization (see Figure 1). The direct prediction effects of emotional creativity on anxiety, depression, and somatization were not significant. In contrast, the direct prediction effect of emotional creativity on PTG was significant. Therefore, we concluded that the last four models were complete mediating models, while the first model was a partial mediating model. Moreover, based on 5,000 bootstrap samples, bootstrap analysis showed that the total indirect effect between emotional creativity and PTG [β = 0.256, SE = 0.053, 95% CI = (0.159, 0.369)], emotional creativity and anxiety [β = −0.248, SE = 0.042, 95% CI = (−0.335, −0.171)], emotional creativity and depression [β = −0.234, SE = 0.039, 95% CI = (−0.317, −0.162)], and emotional creativity and somatization [β = −0.176, SE = 0.038, 95% CI = (−0.255, −0.105)] were significant. All mediating effect estimations based on the non-parametric bootstrap method are shown in Table 2. The results indicated that both perceived social support and regulatory emotional self-efficacy mediated the effects of emotional creativity on PTG, anxiety, and depression, while only perceived social support mediated the effect of emotional creativity on somatization.

**TABLE 2:** The estimates of indirect effects and the 95% confidence intervals of the estimates (all estimates are under the control of COVID-19-related life events and age).

Indirect effect	β	SE	95% LLCI	95% ULCI
EC→PSS→SRG	0.135	0.043	0.057	0.223
EC→RES→SRG	0.099	0.037	0.029	0.180
EC→PSS→RES→SRG	0.021	0.009	0.007	0.042
Model 1 total indirect effect	0.256	0.053	0.159	0.369
EC→PSS→ANX	−0.177	0.032	−0.238	−0.116
EC→RES→ANX	−0.058	0.032	−0.129	−0.001
EC→PSS→RES→ANX	−0.012	0.007	−0.030	−0.001
Model 2 total indirect effect	−0.248	0.042	−0.335	−0.171
EC→PSS→DEP	−0.164	0.029	−0.224	−0.111
EC→RES→DEP	−0.058	0.031	−0.124	−0.002
EC→PSS→RES→DEP	−0.012	0.007	−0.029	−0.001
Model 3 total indirect effect	−0.234	0.039	−0.317	−0.162
EC→PSS→SOM	−0.138	0.028	−0.197	−0.085
EC→RES→SOM	−0.032	0.028	−0.088	0.022
EC→PSS→RES→SOM	−0.007	0.006	−0.021	0.004
Model 4 total indirect effect	−0.176	0.038	−0.255	−0.105

## Discussion

In this study, we found that during the COVID-19 pandemic, individual emotional creativity was significantly positively correlated with individual perceived social support, regulatory emotional self-efficacy, and PTG, while it was significantly negatively associated with individual mental health problems (e.g., anxiety, depression, and somatization), which is consistent with previous studies (Averill, 1999; Lattifian and Delavarpour, 2012; Wang and Yan, 2017).

This study verified that the predictive effects of emotional creativity on PTG were partly mediated by perceived social support, regulatory emotional self-efficacy, and perceived social support and regulatory emotional self-efficacy altogether; predictive effects on anxiety and depression were all mediated by perceived social support, regulatory emotional self-efficacy, and perceived social support and regulatory emotional self-efficacy altogether; and predictive effects on somatization were all mediated by perceived social support. Our findings correspond with the research of Orkibi and Ram-Vlasov (2019) that emotional creativity can positively predict PTG and negatively predict mental health problems. Moreover, Orkibi and Ram-Vlasov (2019) found that creative self-efficacy mediated the positive association between emotional creativity and PTG as well as the negative association between emotional creativity and mental health problems. In this study, we found that regulatory emotional self-efficacy may play a similar role.

In terms of the intermediary model, perceived social support is a subjective feeling of social support. Individuals with high emotional creativity can take deep consideration of the emotions and behaviors of other people and better tolerate emotional conflicts within themselves or others (Averill and Thomas-Knowls, 1991; Sun and Lu, 2009). Therefore, on the one hand, individuals with high emotional creativity may be better at exploring emotional support given by others; on the other hand, these people are also more likely to establish emotional connections with others because of their effectiveness and sincerity in emotional expressions. Social support from others, as a supplement to relieve emotions or stress, essentially constitutes an emotional adjustment resource for individuals. For example, some of the items in the MSPSS involve emotional support from the people around (e.g., “There is a special person with whom I can share my joys and sorrows” and “I get the emotional help and support I need from my family”). Individuals who know that they have such resources may be more confident in emotional adjustment, thereby improving their regulatory emotional self-efficacy, ultimately promoting their PTG and improving their mental health.

In terms of negative posttraumatic outcomes, we found that although anxiety, depression, and somatization were all mental health problems, the internal mechanisms of emotional creativity in predicting them were not the same. A possible explanation is that anxiety and depression are highly related to emotions, while somatization is also emotionally related, but it is more self-imperceptible; therefore, the mechanisms of predictive effect are different. Another theory may be needed to explain how emotional creativity affects somatization.

This study explored the impacts and mechanisms of emotional creativity on individual posttraumatic outcomes in grave public health events and provided theoretical and empirical support regarding the impact of emotional creativity on individual stress responses, making contributions to understanding the relationship between emotional creativity and posttraumatic outcomes and supporting the social construction theory of emotion. Besides, research in the context of magnitude of public health events, for one thing, can compensate for the deficiency of most previous studies on PTG that were limited to a certain kind of disease or early traumatic experiences. Moreover, it complements the relevant evidence of influencing factors and mechanisms of PTG in the case of disastrous outbreaks. Therefore, the study attested that emotional creativity can reduce mental health problems caused as a result of grave public health emergencies and bring more PTG to individuals, suggesting that researchers can help citizens keep physically and mentally healthy during the period of grave public health emergencies by improving their emotional creativity. At present, the COVID-19 pandemic is still rampant worldwide; therefore, it is of great clinical significance to explore solutions to the pandemic.

There are several limitations to this study. First, the sample size of this study was small, and we used convenience sampling, which may have affected the statistical power and external validity of this study. In the future, we expect to expand the sample size and improve the sampling method based on this study to improve the statistical power and external validity. Second, this study only discusses the chain-mediating role of perceived social support and regulatory emotional self-efficacy as intermediary variables. It still needs further explorations to find out whether other variables affect the process and whether the mediating variables are regulated by other variables. Third, this study is a cross-sectional study. Thus, the results only confirm the relationship between the variables at the relevant level. In the future, researchers can explore the relationships among variables on a larger time scale, taking a cross-lagged design, longitudinal study, or other methods to further determine the relationship among variables and the possible dynamic process.

## Data Availability Statement

The raw data supporting the conclusions of this article will be made available by the authors, without undue reservation.

## Ethics Statement

The studies involving human participants were reviewed and approved by Institutional Review Board of Psychology of the Nankai University. The patients/participants provided their written informed consent to participate in this study.

## Author Contributions

H-KZ, Y-XH, Y-XC, X-WW, QL, and XZ developed the research idea together. Under the supervision of XZ and QL, H-KZ, Y-XC, and X-WW designed the questionnaire and collected the data for this article. H-KZ analyzed the data. QL, H-KZ, and Y-XH drafted the manuscript. XZ, H-KZ, Y-XH, and X-WW provided critical revisions. All authors contributed to the article and approved the submitted version.

## Conflict of Interest

The authors declare that the research was conducted in the absence of any commercial or financial relationships that could be construed as a potential conflict of interest.
